# Non-adherence to medication and associated factors among type 2 diabetes patients at Clinique Medicale Fraternite, Rwanda: a cross-sectional study

**DOI:** 10.1186/s12902-022-01133-0

**Published:** 2022-08-31

**Authors:** Jean de Dieu Murwanashyaka, Albert Ndagijimana, Emmanuel Biracyaza, François Xavier Sunday, Maryse Umugwaneza

**Affiliations:** 1 Department of Epidemiology and Biostatistics, School of Public Health, University of Rwanda, Kigali, Rwanda; 2 Programme of Sociotherapy, Prison Fellowship Rwanda (PFR), Kigali, Rwanda; 3 College of Medicine and Health Sciences, University of Rwanda, Kigali, Rwanda

**Keywords:** Diabetes Mellitus, Adherence, Prevalence, Medication, Prescribed

## Abstract

**Background:**

Type 2 Diabetes Miletus (T2DM) is a public health burdens that alarmingly increases and leads to morbidity and mortality over the last decades globally. Its management is multifaceted and adherence to diabetic medications plays great roles in life of T2DM patients. But epidemiology on adherence and its associated factors remain unknown in Rwanda. Therefore, this study determined the extent of non-adherence and its predictors among T2DM patients seeking healthcare services at the Clinique Medicale la Fraternite.

**Methods:**

A cross-sectional study among 200 adults’ patients with T2DM receiving care in the Medicale la Fraternite clinic was investigated. Bivariate and multivariate logistic regression models were performed based on odds ratio employed to examine associated predictors of non-adherence. The cut-off value for all statistical significances tests were considered at *p* < 0.05 with 95% for the confidence intervals.

**Results:**

Overall, more than a half of T2DM patients (53.5%) had poor medication adherence. Being females [OR = 2.1, 95%CI(1.13–3.71), *p* = 0.002], consuming anti-diabetic drugs for 4–10 years [OR = 2.18, 95%CI(1.09–4.34), *p* = 0.027], experiencing poor communication with healthcare providers [OR = 2.4; 95%CI (1.36–4.25), *p* = 0.003] and being perceived as burden of the family [OR = 5.8; 95%CI(1.3–25.7), *p* < 0.021] had higher odds of non-adherence to anti-diabetic medications. Those with poor HbA1C [OR = 4.26; 95%CI(1.7–10.67), *p* = 0.002] had 4.26 times higher odds to be non-adherent compared to those with good HbA1C. Respondents with primary [OR = 3.56; 95%CI (1.12–11.28), *p* = 0.031] and secondary education [OR = 2.96; 95%CI (1.11–7.87), *p* = 0.03] were more likely to be non-adherent than those with informal education respectively. Those with normal BMI [OR = 5.17; 95%CI(1.63–16.37), *p* = 0.005] and those with overweight or obese [OR = 3.6; 95%CI (1.04–9.1), *p* < 0.02] had higher odds of being non-adherent than those with underweight.

**Conclusion:**

Sex, glycaemia, communication with healthcare providers, education and gycosylated hemoglobin were the major predictors of non-adherence. Interventions for tackling this problem through bringing together efforts to stem this epidemic and controlling predictors of non-adherence are urgently recommended.

## Background

Type II Diabetes Mellitus (T2DM) refers to a group of heterogeneous disorders with the common elements of chronic hyperglycemia and glucose intolerance due to insulin deficiency, impaired effectiveness of insulin action, or both [1, 2]. The prevalence of T2DM continues increasing dramatically in developed and developing countries [2, 3]. This disease accounts for approximately 90% of all patients (347 million cases globally) with diabetes worldwide [4–6]. Although approximately 4% of overall global deaths of T2DM occur, more than 80% of mortality occurs in the low-and middle-income countries (LMICs) [7, 8]. Additionally, the management of this chronic metabolic disease incurs remarkable a burden due to its diverse complications and outcomes largely attributed to non-adherence to anti-diabetic medications that remain a concern [9, 10]. The World Health Organization (WHO) projected that by 2040, 8.5% of the world’s population (more than 642 million patients with diabetes) will have T2DM in comparison to 4.7% in 1980s [11, 12]. Between 2010 and 2030, the number of adults is expected to rise up to 69% in developing countries and by 20% in industrial countries of the world [7, 13]. Thus, there is an increase of biopsychophysiological conditions as outcomes of T2DM and negative attitudes (such discrimination, social rejection, blame, stigmatise, discouraging patients) of the family members towards their T2DM patients. These attitudes may lead to worse health outcome for the patients or cause feelings of fear, embarrassment, anxiety, low self-esteem, guilt and blame [14, 15].

In the case that patients with T2DM do not adhere to medications, serious effects may arise, such as disease relapse and progression [16]. In Sub-Saharan African (SSA) countries such as in Uganda, Burundi, Tanzania and Kenya, non-adherence to anti-diabetic drugs is debatable and a continuation of this concern was documented [17, 18], if no preventive measures are not taken for decreasing its factors [19–24]. Rwanda has experienced an increase of the morbidity of the diabetes and this has reached to more than 2% among overall deaths [25]. Besides, T2DM is caused by behavioral influences compromising poor diet, lack of physical exercises, obesity, use of alcohol, and drugs. Although almost factors are related to lifestyle habits of the individuals, environmental influences (internal environmental factors like inflammatory and adipocytokines and hepatocyte factors; external environmental factors like endocrine disruption) and genetic influences are established [26, 27]. Other contributing factors to T2DM include fat distribution, family history, pre-diabetes and gestational diabetes [20, 28].

Adherence to anti-diabetic medications refers to the extent to which an individual’s medication use behaviour coincides with medical advice, and persistence as the duration of time from initiation to discontinuation of therapies [29, 30]. The WHO in its landmark report on non-adherence globally estimated 50% adherence to T2DM medications and further stated that adherence was more likely to lower in developing countries owing to resource limitations [12, 31, 32]. Thus, non-adherence to anti-diabetic medications varies from 36 to 93% [33, 34] and this can alter the level of blood glucose and causes the complications that weaken the quality of life [35]. Further, more than a half of patients with T2DM have poor glycemic control [36] and this burden exacerbate life in the countries from East Africa like Ethiopia [35], Tanzania, Uganda and Kenya [37, 38]. More, high cost of anti-diabetic drugs, long duration of taking medications, side effects of anti-diabetic drugs such as fatigue, nausea, vomiting, psychological problems and itching, socio-cultural issues, substance use, health literacy, belief, availability and affordability of insulin, and non-persistence are factors of non-adherence [7, 39, 40]. Previous studies identified factors of non-adherence such as poorer treatment outcomes, low demographic characteristics (like young age, lower education and income), progression of disease symptoms and complications [10, 28]. Optimal glucose control can be achieved through strict compliance to medications, diet, and lifestyle modifications, which in turn minimize long-term complications [41–44]. This results in encountering difficulties to maintain the desired level of required glycaemia [45–47].

Earlier studies agreed that the adherence to anti-diabetic drugs plays crucial roles in promoting quality of life of patients and this prevents long-term harmful effects [48, 49]. In the region of East Africa, socio-demographic and behavioral problems were reported as factors contributing to adherence to anti-diabetic drugs. For example, in Ethiopia, Kenya and Tanzania stated that the prevalence of non-adherence varies from 17.5% to 28.9% [50, 51]. Among the factors leading to non-adherence to anti-diabetic medications, types of medications were documented [40, 52]. For instance; patients under insulin experience non-adherence because medications of insulin and its available remain challenge in most countries when compared to OHA [53, 54]. So, non-adherence to T2DM drugs was described as a product of the interaction between patient-related factors (like demographic and psychosocial factors), disease-related factors (such as comorbidities, blood glycose monitoring and complications) and medical system [55, 56].

According to several previous studies, better adherence with older patients while others reported younger patients as being more adherent to medications [37, 57]. Further, psychological influence on adherence where patients with diabetes with stigma, depression, eating disorders, and anxiety develop a higher risk to poor adherence to medications [8, 58]. Pertaining to diabetics, treatment-related barriers and negative emotions contribute to lack of success in reducing HbA1C [59, 60]. In addition, problems with adherence are cyclical in that poor adherence may lead to poor glycemic control, which creates treatment-oriented frustration [38, 61]. Moreover, non-adherence is influenced by the factors like the number of medications (pills burden), cost for medications, side effects, dosage frequency, dosage regimens [62–64]. Hence, having regular and frequent contact with patients by telephone promoted regimen adherence and achieved improvements in glycemic control, as well as in lipid and blood pressure levels contribute to adherence [56, 65].

The Diabetes Atlas reported expected dramatic increase of T2DM from 3.2% and this makes it one of national pestilence [25, 66]. Non-adherence to prescribed anti-diabetic drugs is also controversial and there is a need to attenuate the factors contributing to non-adherence to drugs [67]. Though the non-adherence remains a public health concern in Rwanda, there is a gap in knowledge about prevalence of non-adherence to anti-diabetic medications and its predictors in Rwanda. With this background and gap, this study determined the prevalence of non-adherence to anti-diabetic medications and its associated predictors in the Clinique Medicale Fraternite. The results of this study are expected to guide the development of effective and customized strategies to enhance patients’ medication adherence and improve their diabetes outcomes in the long term.

### Conceptual framework

**Fig. 1:**
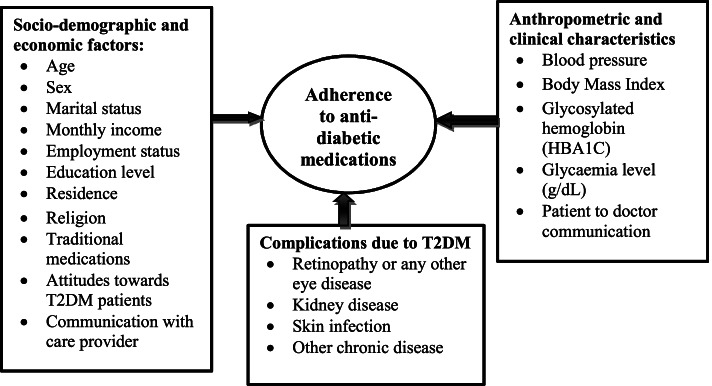
Conceptual framework. Abbreviations: BMI: Body Mass Index, HbA1C: Glycosylated haemoglobin, T2DM: Type 2 Diabetic Mellitus, g/dL: gram per decilitre; kg/m^2^: Kilogram per square metre

Our conceptual framework developed based on the factors associated with non-adherence in T2DM patients that were documented in previous epidemiological studies. Those factors compromise socio-economic and demographic factors (such as age, sex, marital status, monthly income, employment status, education, residence, religion, traditional medications, attitudes towards T2DM patients (such as stigmatisation, social rejection, discouraging them, blame, feeling concerned in disease management, feeling well concerned, feeling that the T2DM patient is the burden for the family), patient to doctor communication); anthropometric and clinical characteristics (such as blood pressure, Body Mass Index, glycosylated haemoglobin, glycaemia level); and complications due to T2DM, retinopathy or any other eye disease, kidney disease, skin infection and other chronic disease) (Fig. 1).

## Methods

### Study design and settings

Institutional cross-sectional study design was conducted among 200 hospitalized and ambulatory patients diagnosed T2DM at the Clinique Medicale Fraternite. Clinique Medicale Fraternite is the clinic Kigali city, capital city of Rwanda and specialized in providing healthcare among patients with diabetes in Rwanda. This clinic was found in 1997 and serves as one of important health facilities for diabetes in Rwanda. It is also the only national specialized diabetes clinical in Rwanda as established by the Rwandan Diabetes Association (RDA). Its staff is composed of doctors and nurses who work for providing healthcare for the patients with diabetes. More than 12,000 patients have been screened for diabetes at the clinic which has its own laboratory with an HbA1C machine. More than 6000 patients with diabetes are currently registered at the clinic, while others follow treatment in the hospitals. The clinic has six beds and a trained diabetes nurse present 24 h [66]. It also operates from 24 h daily and delivers the anti-diabetic treatments and medical follow-up for the patients with T2DM.

### Study population and sample size

The participants were patients diagnosed and treated T2DM. Our study enrolled adult patients, diagnosed and confirmed as patient at least 6 months prior to the study start, and those with willingness and ability to participate. All eligible participants were registered by the RDA. The exclusion criteria were having several health conditions including cognitive impairments, not providing written consent forms for participating, having severe hearing and visual impairments, and lack of voluntariness in participating. Then, sample size was calculated using single proportion formula [68] considering a 95% (z = 9.16) confidence intervals, 5% margin error (e = 0.05) and a known prevalence of non-adherence (*p* = 34% or 0.34) to diabetic medication among patients from surrounding country of Rwanda [69]. This resulted in a calculated sample size of 336 patients.

$${\varvec{n}}=\frac{{1.96}^{2}*0.34(1-0.34)}{{0.05}^{2}}=336$$

### Data collection and sampling techniques

A convenient sampling technique was employed for participant recruitment. Data collectors were provided the list of the potential participants and when the patient came at the appointment was given the list to check if they were eligible to participate. Then, the interested participants who accepted voluntarily to participate in the study were given a copy of their current medication list record and then referred to the research for taking part in the interview. Before interview, the researcher brought the respondents in the private area for explaining to them about the research. Further, data collection was performed from March 1, 2019 to September 30, 2019 by trained data collectors whose educational background was nursing and public health. After a short term-training deliver to the data collectors, data collection was conducted under coordination of the authors. Data were collected from the diabetic clinic day when the patients came for seeking health interventions. Participants completed the entire survey between 25 and 45 min.

#### Socio-demographic characteristics and clinical data

Socio-demographic questionnaire included variables such as age, residence, sex, employment status, religion, marital status, monthly income, education, use of traditional medications and attitudes towards patients with T2DM.

#### Patient-Doctor Communication Inventory (PDCI)

The PDCI is the 7-item psychometric instrument used to assess the level of communication between the doctor and patient [70, 71]. This measurement was designed since communication has a prominent contribution to patient satisfaction and adherence to medication prescribed by the healthcare provider. Communication also helps the patient to understand the diagnosis and treatment that also has an impact on care, have a relevant expectation and involvement in their treatments [72]. This instrument is mostly used in medication settings and has the items scored using 5-point Likert Scale (1 = never to 5 = always). After scores, each item the total scores are calculated and then are averaged into a single synthetic score reflecting overall performance. In our studies, PDCI has a satisfactory internal consistency (Cronbach’s Alpha, α = 0.89) as it is described in the previous studies [71].

#### Morisky-Green-Levine Scale (MGLS)

In the present study, the four-item Morisky Medication Adherence Scale (MGLS), which includes four questions with yes/no response options was used to measure the level of the adherence to anti-diabetic medication [73, 74]. The total scores of this instrument vary from 0 to 4. This MGLS is the medical measurement scored using bivariate (0 = no, 1 = yes) to assess the level of the adherence to medical drugs. The score of 4 indicated the high level of adherence [75]. The MGLS results also developers suggested three levels of adherence to the medications based on these scores: high, medium and low adherence with 0, 1, 2, and 3–4 points respectively. Further, the Alpha of Cronbach of this instrument was adequate (α = 0.61). Its cut-off is 3 is used for indicating that those with 3 score or more are non-adhered. The internal consistency of MGLS was adequate [74, 76]. Additionally, the MGLS had a dichotomous definition of adherence based on MGLS is also commonly used with 0 points indicating perfect adherence and 1 + points indicating some level of non-adherence [74, 76, 77]. In our study, MGLS has an acceptable internal consistency (Alpha of Cronbach, α = 0.88).

#### Anthropometric status and clinical data

With the data of height, Body Mass Index (BMI) was performed. Height of the participants was measured without shoes to the nearest of 0.1 cm (cm) using the stand-meter whereas the weight was measured using the nearest of 0.1 kg on a hospital scale with the eligible patients to participate in the study who were wearing one-layer of the clothes and without shoes for avoiding the overestimation. The BMI was computed by taking the weight (kg) divided by the square of the height (m^2^). In this study, three categories of the BMI were demonstrated by using the recommended cut-offs where BMI < 18.5 kg/m^2^ indicated underweight, BMI = 18.5 kg/m^2^-24.9 kg/m^2^ was for patients with normal BMI, BMI ≥ 25 kg/m^2^ indicated overweight whereas BMI ≥ 30 kg/m^2^indicated obesity [78]. Regarding clinical data, data about medication regimen such as number of medications, biomedical (blood pressure, glycosylated hemoglobin, level of glycemia), duration of disease, route of administration, type of medication, dosage frequencies, and complications status were collected. Additionally complications such as diagnosis of retinopathy, eye disease, skin infection, kidney disease, and other chronic disease were clinically collected.

### Data analysis

Statistical analyses were performed using the Statistical Package for Social Sciences (SPSS) version 24. The statistical parameters for descriptive analysis included frequency, means and standard deviation to describe the participants and report the prevalence of non-adherence. In analytical analysis, bivariate and multivariate logistic regressions based on the odds ratio (OR) were employed to determine the associated factors of non-adherence to T2DM medications. Using the Database of this study, the internal consistent reliability using the Alpha of Cronbach was computed to indicate the consistency of the psychometric measures we used. Certainly, the Chi-square test was computed to measure the statistical significance of the association between the outcome and independent variables. The 95% confidence intervals and significance levels of *p* < 0.05 were statistically respected.

### Ethical considerations

All methods were conducted in accordance with the tenets or regulations and principles of Helsinki Declaration [79]. Ethical approval was obtained from the Institutional Review Board, College of Medicine and Health Sciences at the University of Rwanda (N^0^: 460/CMHS IRB/2019). Authors were authorized by the staff of the Clinique Medicale Fraternite to collect data. Also, the permission to use MGLS was sought from Morisky-Green-Levine as required. Confidentiality was ensured. Participants provided the informed consent forms to participate.

## Results

### Socio-demographic characteristics of the participants

Among 336 eligible patients, only 212 (63.1%) participants took part in the study. A completion rate of 94.3% (*n* = 200) was achieved after excluding those whose data were incomplete and those who denied participating in the study. The main reasons for refusing to participate among 124 participants were lack of interest (*n* = 9), lack of time (*n* = 27), not feeling well on the day of the day collection (*n* = 46), complaint of compensation of taking part in the study (*n* = 32), and unknown reasons (*n* = 10). A majority of respondents (59.5%) were aged 50 years and above. The majority of the participants attended primary schools (51%) while 21% had not obtained any formal education. Concerning the employment status, the results of this study found the majority were unemployed (42.5%) whilst 18.5% and 39% worked in public institutions and private sectors respectively. The results represented the majority of the respondents were Christians who represented 74.5% of the sample. The 81.5% of the participants who were enrolled were from Kigali city while 18.5% were from the other settings out of Kigali. About monthly income, majority (52.5%) of the participants received less than 100,000rwf (Table 1).

**Table 1:** Socio-demographic characteristics of the participants

Variables	Frequency	Percentage
**Age**		
18–30 years	10	5
31–50 years	71	35.5
More than 50 years	119	59.5
**Sex**		
Male	94	47
Female	106	53
**Marital Status**		
Single	24	12
Married/cohabiting	145	72.5
Divorce/widowed/widow	31	15.5
**Education**		
No formal education	42	21
Primary	102	51
Secondary/higher/ university	56	28
**Employment status**		
Unemployed	85	42.5
Public sectors	37	18.5
Private sectors and others (e.g., business, farmers, livestock etc.)	78	39
**Religion**		
Christians	149	74.5
Muslim and others	51	25.5
**Residence**		
Gasabo	59	29.5
Nyarugenge	52	26
Kicukiro	52	26
Out of Kigali	37	18.5
**Monthly income**		
< 100000rwf	105	52.5
100,000–200,000rwf	69	34.5
> 200000rwf	26	13
**Patient Doctor Communication Inventory (PDCI)**		
Poor communication	89	44.5
Good communication	111	55.5

### Anthropometric measurements and clinical records of the patients with T2DM

The results indicated that among majority of the participants (57%) had normal blood pressure. Glycemic control as determined by HbA1C was poor (> 7%) for 163 (81.5%) participants who participated in this research while only 18.5% represented good glycemic level. Findings indicated the patients with diabetes had different levels of BMI where majority of them had normal BMI (37%), and only 34% and 19% were overweight and obese. Concerning the medical complications, the results showed that 53% of the patients with diabetes had retinopathy, 26% kidney disease, 40% skin infection and 49% for other chronic diseases. The overall Alpha of Cronbach of DPCI was 0.76 indicating a satisfactory level of internal consistency (Table 2).

**Table 2:** Anthropometric measures and clinical characteristics of the participants

Variables	Frequency	Percentage
**Blood pressure**		
Normal	114	57
Hypertension	86	43
**BMI**		
Underweight (< 18.5)	20	10
Normal (18.5–24.9)	74	37
Overweight (25–29.9)	68	34
Obese (30 and more)	38	19
**Glycosylated Hemoglobin(HbA1C)**		
Good(= < 7%)	37	18.5
Poor (> 7%)	163	81.5
**Glycemia (g/dl)**		
Less than 170 g/dl	33	16.5
170–250 g/dl	47	23.5
More than250 g/dl	120	60
**Presence of diabetes complications**		
***Retinopathy***		
Yes	106	53
No	94	47
***Kidney disease***		
Yes	52	26
No	148	74
***Skin infection***		
Yes	80	40
No	120	60
***Presence of other chronic disease***		
**Yes**	**99**	**49.5**
**No**	**101**	**50.5**

### Attitudes of the family members towards the patients with T2DM

**Fig. 2:**
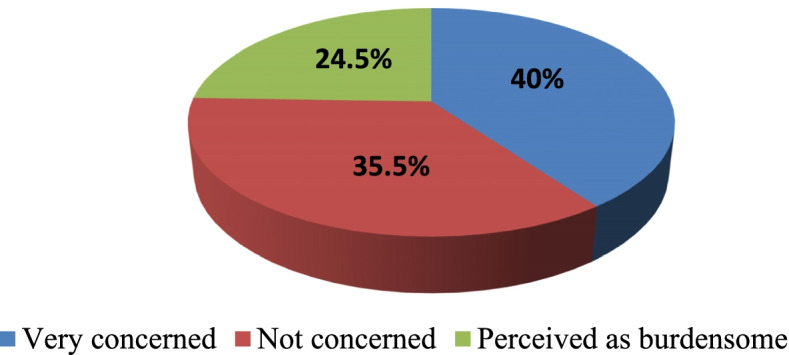
Attitudes of the family members towards to patients with T2DM. Very concerned: Refers to the family members who perceive well the T2DM and remains concerned in management of this disease; Not concerned: Refers to those who are not concerned about the T2DM; Perceived as burdensome: This refers to the family members who consider the T2DM as the burden of the family

The results indicated that the patients who were enrolled in this study were from the family that had different attitudes towards the patients with the T2DM. It was found that the majority (40%) of the patients were from the families whose members were very concerned to the diagnosed diseases and its management. This was followed by 35.5% of those who declared that their family members were not concerned to their disease. But, only 24.5% of T2DM were from the families that perceived them as their burdens or burdensome (Fig. 2).

### Prevalence of medication non-adherence using MGLS

**Fig. 3:**
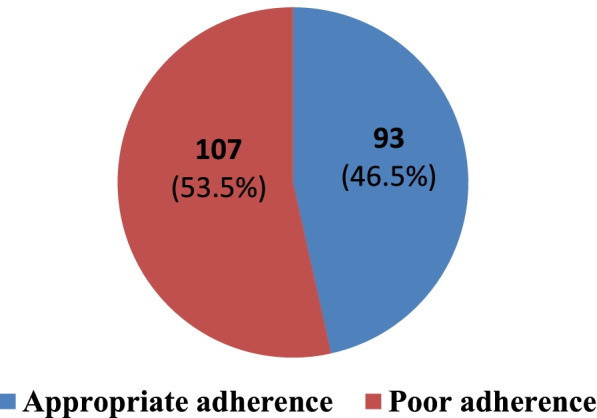
Prevalence of non-adherence to anti-diabetic medications

The prevalence of the adherence among the patients with T2DM using MGLS was calculated and our findings indicated that the prevalence of non-adherence to anti-diabetic medications was 53.5% [95%CI(47–60)], representing 107 patients with diabetes (Fig. 3).

### Association between different factors and non-adherence among the patients with T2DM

Our findings presented significant associated factors of non-adherence such as socio-demographic factors (such as sex, education, employment, and residence of the diabetic patient), clinical records (like BMI, medical drugs, duration of diabetes, glycaemia, DPCS, HbA1C) and diseases complications such as skin infections, and retinopathy. Bivariate analysis indicated the following variables are significantly associated with the non-adherence to anti-diabetic drugs; sex (*p* = 0.032), duration of diabetes (*p* = 0.031), education (*p* = 0.036), employment status (*p* = 0.008), residence of the patient (*p* = 0.021), medical drugs (*p* = 0.027), glycaemia level (*p* = 0.046), BMI (*p* = 0.029), retinopathy (*p* = 0.052), DPCI (*p* = 0.003), skin infection (*p* = 0.035), and HbA1C (*p* = 0.013)(Table 3).

**Table 3:** Bivariate logistic analyses of association between socio-demographic characteristics and non-adherence among patients with T2DM

**Variables**	**Non-adherence**		**Odd ratios, 95% CI**			***P-value***
	**N**	**%**	**OR**	**Lower**	**upper**	
**Age**						
18–30 years	3	2.8	1			0.32
31–50 years	40	37.38	2.71	0.670	11.01	0.16
More than 50 years	64	59.81	0.9	0.499	1.63	0.73
**Sex**						
Male	47	43.93	1			0.032*
Female	60	56.07	1.85	1.05	3.24	
**Marital Status**						
Single	9	8.41	1			0.26
Married/cohabiting	81	75.70	2.02	0.682	6.01	0.204
Divorce	17	15.89	0.96	0.44	2.1	0.917
**Duration with diabetes and medications**						
1-3 years	49	45.79	1			0.031*
4-10yrs	26	24.3	2.18	1.09	4.34	0.027*
More than 10 years	32	29.91	1.03	0.43	2.42	0.954
**Education**						
No formal education	17	15.89	1			0.036*
Primary	51	47.66	2.87	1.27	6.5	0.012*
Secondary	39	36.45	1.834	0.94	3.58	0.075
**Employment status**						
Jobless	28	26.17	1			0.008**
Public employee	28	26.17	2.36	1.24	4.48	0.009*
Private	51	47.66	0.8	0.38	1.72	0.57
**Religion**						
Christian	83	77.57	1			0.482
Muslim	21	19.63	0.477	0.11	2.07	0.323
Other	3	2.80	0.629	0.13	2.97	0.557
**Residence**						
Kigali	76	71.03	1			0.021*
Out of Kigali	31	28.97	2.3	1.14	4.660	
**DPCS**						
Good communication	37	34.58				0.003**
Poor communication	70	65.42	2.4	1.36	4.25	
**Attitudes of the family members towards patients with T2DM**						
Very concerned	46	42.99	1			0.005*
Not concerned	37	34.58	0.5	021	12	0.23
Perceived as burdensome	24	22.43	4.8	1.2	19.8	0.032*
**Monthly income**						
< 100,000rwf	56	52.34	1			0.89
100,001–200,000rwf	36	33.64	1.19	0.5	2.84	0.69
> 200,000rwf	15	14.02	1.25	0.5	3.11	0.63

*N* Indicates number of cases or frequency, *%* Percentage, *OR* Odds ratio, *CI* Confidence Intervals

*p < *0.05*

**p < *0.01*

***p < *0.001*

Our results that the following variables are significantly associated with the adherence to anti-diabetic drugs, medical drugs (*p* = 0.027), glycaemia level (*p* = 0.046), BMI (*p* = 0.029), retinopathy (*p* = 0.052), DPC I(*p* = 0.003), skin infection (*p* = 0.035), and HbA1C (*p* = 0.013)(Table 4).

**Table 4:** Association between non-adherence and anthropometric measures and complications

**Variables**	**Non-adherence**		**Odd ratios, 95% CI**			***P-value***
	**N**	**%**	**OR**	**Lower**	**upper**	
**Anthropometric data**						
**Blood pressure**						
Normal	52	48.6	1			0.672
Hypertension	30	28.04	1.13	.644	1.980	
**BMI**						
Underweight (< 18.5 kg/m^2^)	10	9.35	1			0.029*
Normal (18.5 kg/m^2^-24 kg/m^2^)	32	29.91	1.64	0.88	3.1	0.12
Overweight/obese (> 24 kg/m^2^)	65	60.75	3.01	1.3	7.12	0.01*
**Types of the medical drugs**						
Single OHA	59	55.14	1			0.027*
OHA and Insulin	29	27.1	0.43	0.22	0.86	0.017*
Insulin alone	19	17.76	0.37	0.16	0.84	0.017*
**Glycosylated Hemoglobin (**HbA1C**)**						
Good (= < 7%)	20	18.69	1			0.013*
Poor (> 7%)	87	81.31	2.28	1.19	4.36	
**Glycaemia (g/dl)**						
Less than 170 g/dl	11	10.28				0.046*
170–250 g/dl	28	26.17	1.96	0.86	4.44	0.108
More than250 g/dl	68	63.55	.567	0.27	1.18	0.127
***Presence of diabetes complications***						
**Retinopathy complication**						
Yes	48	44.86	1			
No	59	55.14	0.83	0.45	1.46	0.052
**Kidney disease**						
Yes	77	71.96	1			0.216
No	30	28.04	0.68	0.36	1.26	
**Skin infections**						
Yes	71	66.36				0.035*
No	36	33.64	.541	0.31	0.96	
**Presence of other chronic disease**						
Yes	99	92.52				
No	101	94.39	1.09	0.62	1.9	0.68

*N* Indicates number of cases or frequency, *%* Percentage, *OR* Odds ratio, *CI* Confidence Intervals, *OHA* Oral Hypoglycemic Agents, *DPCS* Doctor- Patient Communication Scale, *BMI* Body Mass Index, *HbA1C* Glycosylated Hemoglobin

*p < *0.05*

**p < *0.01*

***p < *0.001*

### Multivariate logistic regression models for the factors of non-adherence to medications

The results showed that the females were found to have a greater odd to be non-adhered to medications [OR = 2.1, 95%CI = 1.13–3.71), *p* = 0.002] than the males. Those who spent 4-10 years under anti-diabetic drugs were more likely to be non-adherent to medications (OR = 2.5, 95%CI = 1.04–6.37) compared to who spent less than 4 years under anti-diabetic medications. Further, patients who studied primary [OR = 3.56; 95%CI(1.12–11.23), *p* = 0.031] and secondary schools [OR = 2.96; 95%CI(1.11–7.87), *p* = 0.03] were more likely to develop poor adherence to medications when compared to those with no formal education. Respondents with normal BMI had greater odds to be non-adherent [OR = 5.17; 95%CI(1.63–16.37), *p* = 0.005] than those with underweight while those with overweight or obese were almost 4 time more likely to be non-adherent [OR = 3.6; 95%CI (1.04–9.1), *p* < 0.02] than the patients with underweight. Respondents under OHA and insulin medications were less likely to be non-adherent than the patients under single OHA [OR = 0.59; 95% CI(0.24–1.42), *p* = 0.023] and the patients under insulin only were less likely to be non-adherent [OR = 0.26; 95%CI(0.09–0.74), *p* = 0.011] compared to the patients under single OHA. Patients who experienced a poor communication with the healthcare provider were more likely to experience non-adherence to medications [OR = 2.18; 95%CI (1.013–4.69), *p* = 0.046] than their counterparts. Patients with diabetes with poor HbA1C were less likely to be non-adherent [OR = 4.26; 95%CI(1.7–10.67), *p* = 0.002] than their counterparts. Those with 170-250 g/dl of glycaemia were more likely to be non-adherent [OR = 3.17; 95%CI(1.06–9.5), *p* = 0.045] compared to the T2DM who had < 170 g/dl of glycaemia. The patients with skin infections had less chances to be non-adherent [OR = 0.36; 95%CI(0.17–0.77), *p* = 0.009] than their counterparts (Table 5).

**Table 5:** Multivariate analysis for the associated factors of the non-adherence to medications

**Variables**	**Non-adherence**		**95%CI**			***P-value***
	**N**	**%**	**OR**	**Lower**	**upper**	
**Sex**						
Male	47	43.93	1			0.002**
Female	60	56.07	2.1	1.13	3.71	
**Duration of diabetes**						
1-3 years	49	45.79	1			0.042*
4-10yrs	26	24.3	2.57	1.04	6.37	0.023*
More than 10 years	32	29.91	1.03	0.34	3.12	0.031*
**Education**						
No formal education	17	15.89	1			0.002*
Primary	51	47.66	3.56	1.12	11.28	0.031*
Secondary	39	36.45	2.96	1.11	7.87	0.030*
**DPCS**						
Good communication	37	34.58	1			0.046*
Poor communication	70	65.42	2.18	1.01	4.68	
**Employment status**						
Jobless	28	26.17	1			0.061
Public employee	28	26.17	2.01	0.92	4.38	0.08
Private	51	47.66	1.06	0.38	2.97	0.906
**Residence**						
Kigali	76	71.03	1			0.089
Out of Kigali	31	28.97	2.19	0.89	5.39	
**Attitudes of the family members towards patients with T2DM**						
Very concerned	46	42.99	1			0.031*
Not concerned	37	34.58	0.1.5	0.4	5.5	0.54
Perceived as burdensome	24	22.43	5.8	1.3	25.7	0.021*
**BMI**						
Underweight (< 18.5 kg/m^2^)	10	9.35	1			0.003**
Normal (18.5 kg/m^2^-24 kg/m^2^)	32	29.91	5.17	1.63	16.37	0.005*
Overweight/obese (more than 24)	65	60.75	3.6	1.04	9.1	0.02*
**Anti-diabetic medication**						
Single OHA	59	55.14	1			0.011*
OHA and Insulin	29	27.1	0.59	0.24	1.42	0.023*
Insulin alone	19	17.76	0.26	0.09	0.74	0.011*
**Glycosylated Hemoglobin (HbA1C)**						
Good (= < 7)	20	18.69	1			0.002**
Poor (> 7)	87	81.31	4.26	1.7	10.69	
**Glycaemia (in g/dl)**						
Less than 170 g/dl	11	10.28	1			0.0456*
170–250 g/dl	28	26.17	3.17	1.06	9.5	0.04*
More than250 g/dl	68	63.55	0.8	0.33	1.97	0.626
***Presence of diabetes complications***						
**Retinopathy complication**						
Yes	48	44.86	1			0.325
No	59	55.14	1.43	0.7	2.9	
**Skin infections**						
Yes	71	66.36	1			0.009**
No	36	33.64	0.36	0.17	0.77	

*N* Frequency, *%* Percentage, *OR* Odds ratio, *CI* Confidence Intervals, *OHA* Oral Hypoglycemic Agents, *DPCS* Doctor- Patient Communication Scale, *BMI* Body Mass Index, *HbA1C* Glycosylated Hemoglobin, *rwf* Rwandan francs

*p < *0.05*

**p < *0.01*

***p < *0.001*

## Discussion

The current study determined the prevalence of non-adherence to anti-diabetic medications and its associated factors among T2DM patients at Clinique Medical Fraternite. This study demonstrated that about 53.5% of the participants had non-adherence to medications. This result is similar to the study that was conducted in Cameroun [40].Perhaps this similarity was because the study design used in our study is similar to the study design used in this study. Also, our results stated that the prevalence on non-adherence is higher than the prevalence documented in the previous studies in the Switzerland, Tanzania, Ethiopia, and Kenya [31, 37, 39, 69], however, one few studies reported a higher prevalence of non-adherence to diabetic medication in Ethiopia than ours [17].

In coincide with the elsewhere studies that indicated that the socio-demographic characteristics, medical complications, and the anthropometric records of the T2DM patients predict the non-adherence [31, 38], our results revealed that the sex of the patients with diabetes is a significant predictor of non-adherence where the females were more likely to be non-adhered to medications. Our results also collaborated with the previous studies conducted on the factors associated with non-adherence to anti-diabetic drugs [17]. On the other hand, the proportion of non-adherence in our study was much higher than their counterparts. Our results are in accordance with preceding studies done in Kenya, Ethiopia, Uganda, and Tanzania [17, 37, 39]. Those with secondary school education and university were almost more likely to experience non-adherence to medications when compared to those with no formal education. These findings were corresponding to the studies conducted in SSA such as in Uganda [31, 38]. Further, patients who took anti-diabetic drugs for 4–10 years and more than 10 years were more likely to experience non-adherence to medication when compared to those under anti-diabetic medications for less than 4 years. These results of our study are compatible to the prior studies [37], however, these results contradicted a study conducted Ethiopia and indicated that duration of diabetes, duration of treatment, taking of traditional medicine, other medications for other long term illness, and side effects of the drugs were not significantly associated with non-adherence to diabetic treatment agent [69, 80]. Then, poor social supports such as the supports from the family members, poor family relationship, and domestic violence are important predictors of non-adherence [81].

In our study, the T2DM patients who received combination of OHA and insulin drugs, and who received insulin had less odds of being non-adhered to medications as compared to those who took single. Additionally, our findings revealed that the T2DM whose family members considered them as their burdens were at greater risk to be non-adherent to anti-diabetic drugs when compared to those whose family members are very concerned in the management of the disease and its impact on their life. Most of these patients develop a worse life and became stigmatized. These results are supported by previous studies conducted on the prevalence and factors of non-adherence among T2DM patients [37, 82], despite, a study in Ethiopia challenged these findings [40]. This is because while availability and affordability of insulin remains the challenge that leads to an increase of non-adherence [40, 52], in Rwanda this medication is available and its cost is affordable. Further, participants with poor communication with their healthcare providers more likely experience non-adherence as compared to those with appropriate communication with their healthcare providers. These findings are in line with a study documented Uganda, Tanzania, Kenya, Ethiopia, in Bharat, Portugal and Kingdom of Saudi Arabia [48]. Although our bivariate reported a significant association between non-adherence and the residence, the multivariate analysis revealed no significant association between non-adherence and residence. Our results are not in accordance with the previous studies that documented that diabetes patients from the rural settings are more likely to non-adherence as compared to the urban [38, 51, 83]. Indeed, our results revealed that skin infection increases the odds to present non-adherence when compared to the patients with no skin infection. Our results are relevant to previous studies carried out and indicated the risk factors of non-adherence [17, 48].

This study has several limitations. First, it was primarily limited to a design, cross sectional study design, which was not able to determine the causal relationship for the non-adherence; however, this study was the first to report contributing factors of non-adherence among the T2DM patients. Second, the conception of adherence is large and it has several contributing factors (such as dietary the concept of adherence is broad since it includes the variables like dietary, health literacy, physical exercise, medications). Although, these variables are important contributing factors, we were not able to collect them which could cause underestimation or overestimation between the associated factors of non-adherence. Third, the study was conducted in one institution located in the urban area, Kigali city, while the T2DM patients from rural areas were not considered in this study. Thus, this could limit its generalisability; however, the sample size was large enough to assess the expected differences and associations between variables. So, we recommend further research on experimental study for determining the causal conclusion or indicating the effect of demographic, clinical and psychosocial factors on medical adherence over time to understand the long-term effects. However, these limitations, some strengths warrant discussion. First; this is the first study conducted to indicate the prevalence of non-adherence and its factors among T2DM at Clinique Medicale Fraternite. Second; the present study utilized validated instruments to assess the level of adherence and its associated factors.

## Conclusion

More than a half (53.5%) of T2DM patients was non-adherent to their anti-diabetic medications. Different contributing factors were identified such as sex, education, BMI, type of medications, glycaemia level, attitudes of family members toward the T2DM patients, duration of disease, HbA1C, skin infections, and poor communication with the healthcare providers. Tackling this health concern of non-adherence should be a priority agenda for policy makers and researchers who can have a great contribution to a reduction of the associated factors through collaboration with healthcare providers and pharmacists. Thus, our results should be used a baseline information to the policy makers, concerned governmental bodies such as Ministry of Health and different stakeholders that workaround rational medicine use to assess the currently available policies regarding rational use of medicines. Health facility is recommended to provide health education and counseling to the T2DM patients targeting the patients with suboptimal adherence for intensified adherence counseling which is essential for promoting health of patients with diabetes. More efforts is needed to increase adherence to medication by training healthcare providers about an effective communication with the patients and health education to reinforce patients in knowing full benefits of prescribed therapies. Further study on psychosocial and cultural influences on non-adherence, and prospective study design for determining the risk factors of non-adherence are recommended.

## Data Availability

The datasets generated during and analyzed are available from the corresponding author upon reasonable request. As one of the research questionnaires “Morisky-Green-Levine Scale (MGLS)” designed by Morisky is shared upon the approval of its founder, the dataset is not publicly available.
